# Starvation Alters Gut Microbiome in Black Soldier Fly (Diptera: Stratiomyidae) Larvae

**DOI:** 10.3389/fmicb.2021.601253

**Published:** 2021-02-16

**Authors:** Fengchun Yang, Jeffery K. Tomberlin, Heather R. Jordan

**Affiliations:** ^1^Department of Entomology, Texas A&M University, College Station, TX, United States; ^2^Department of Biological Sciences, Mississippi State University, Starkville, MS, United States

**Keywords:** starvation, functional prediction, total RNA, viable microbiomes, black soldier fly

## Abstract

Unlike for vertebrates, the impact of starvation on the gut microbiome of invertebrates is poorly studied. Deciphering shifts in metabolically active associated bacterial communities in vertebrates has led to determining the role of the associated microbiome in the sensation of hunger and discoveries of associated regulatory mechanisms. From an invertebrate perspective, such as the black soldier fly, such information could lead to enhanced processes for optimized biomass production and waste conversion. Bacteria associated with food substrates of black soldier fly are known to impact corresponding larval life-history traits (e.g., larval development); however, whether black soldier fly larval host state (i.e., starved) impacts the gut microbiome is not known. In this study, we measured microbial community structural and functional shifts due to black soldier fly larvae starvation. Data generated demonstrate such a physiological state (i.e., starvation) does in fact impact both aspects of the microbiome. At the phylum level, community diversity decreased significantly during black soldier fly larval starvation (*p* = 0.0025). Genus level DESeq2 analysis identified five genera with significantly different relative abundance (*q* < 0.05) across the 24 and 48 H post initiation of starvation: *Actinomyces*, *Microbacterium*, *Enterococcus*, *Sphingobacterium*, and *Leucobacter*. Finally, we inferred potential gene function and significantly predicted functional KEGG Orthology (KO) abundance. We demonstrated the metabolically active microbial community structure and function could be influenced by host-feeding status. Such perturbations, even when short in duration (e.g., 24 H) could stunt larval growth and waste conversion due to lacking a full complement of bacteria and associated functions.

## Introduction

Sensations of hunger are caused by an imbalance between energy intake and expenditure (Friedman et al., 1999). One immediate response to hunger is assumed to be the drive to increase food consumption. Malnutrition, on the other hand, is not as simple as hunger, and is defined as either inadequate or excessive consumption of dietary substances ultimately leading to the development of undernutrition or obesity, respectively, and their corresponding health sequelae (Elia, 2017). Though malnutrition is not the same as hunger, they may be connected (Friedman et al., 1999; Elia, 2017).

Recent studies have shown evidence of commensal microorganisms playing significant roles in influencing nutritional decisions, digestion, and metabolism (Read and Holmes, 2017). Many of these organisms have coevolved within their host to perform a number of functions the host would otherwise be unable to accomplish on its own. For instance, host-associated bacteria rapidly adapt to changes in host diet through changes in population and induction of signaling compounds and degradation enzymes that facilitate digestion through absorption and metabolism of complex molecules (Read and Holmes, 2017). Characterization (e.g., structure and function) of bacteria is therefore important for understanding the comprehensive physiology of the gastrointestinal tract microbiota and its relationship to both hunger and malnutrition.

Given their ease in rearing and manipulation, insects offer great benefits as model hosts for studying these processes. They represent the most diverse group of known organisms, and account for approximately 80% of Earth’s animal species (IISE, 2011). Insects also occupy most habitats, and provide natural ecosystem services often taken for granted, such as herbivory, food for animals, and pollination. Additionally, many insects drive ecosystem processes such as nutrient recycling and decomposition.

Studies have investigated the impact of commensals, as well as gut microbiomes on nutrient absorption, brain function, behavior, overall health, and the consequences of diet on insect associated microbiomes (Feldhaar, 2011). However, very few have analyzed data from nutrient deprivation or starvation, and a literature review at the time this manuscript was prepared revealed only one study investigating the effects of prolonged nutrient deprivation on insect gut microbiota (Tinker and Ottesen, 2016). These studies will be important for our comprehensive understanding of how change in nutrient status impacts insect and other hosts’ gut microbiomes, and subsequent changes in insect-microbe functional processes.

The black soldier fly, *Hermetia illucens* (L.) (Diptera: Stratiomyidae) is a well-known, non-pest fly species that, along with associated microbes, converts decomposing matter into proteins and lipids (Diener et al., 2009; Yu et al., 2011). Black soldier fly larvae undergo a fast-growing period and have a huge appetite. Individual black soldier fly eggs can develop into prepupae in about 3 weeks (Tomberlin et al., 2002 and unpublished data). Black soldier fly larvae consume a wide range of organic matter, such as animal manure (Sheppard et al., 1994; Newton et al., 2005; Zhou et al., 2013) and restaurant waste (Nguyen et al., 2015), and transform them into high quality protein approved for use in the aquaculture industry as feed (Bondari and Sheppard, 1987). Furthermore, data demonstrate black soldier fly larval digestion reduces pathogens from within the waste (Erickson et al., 2004; Liu et al., 2008; Lalander et al., 2013, 2015). To date, researchers have shown that black soldier fly larvae can reduce *Escherichia coli* O157:H7 and *Salmonella* Enteritidis in chicken manure (Erickson et al., 2004; Liu et al., 2008; Lalander et al., 2013, 2015), *E. coli* in dairy manure (Liu et al., 2008), and *Salmonella* spp. in human feces as well as in mixed organic wastes (Lalander et al., 2013, 2015). Consequently, industrialization of black soldier flies has been proposed as a means to sustainably convert organic waste (i.e., animal manure or food waste) to protein.

Although the industry is evolving and growing rapidly, many aspects of the basic biology of this species are still unclear. Some studies determined black soldier fly larval gut microbial community structure was significantly influenced by the type of the food consumed (Jeon et al., 2011; Zheng et al., 2013). Other studies demonstrated inoculation of either gut bacteria (Yu et al., 2011) or probiotics (Zheng et al., 2012) into waste could improve larval growth or waste conversion. However, the mechanism of how microbes influence the nutrient uptake ability of the larval host is unclear. Also, the system has not been optimized for converting waste to protein, and reliance on multiple or single batch feedings as a means to produce black soldier flies can result in different levels of production and waste conversion (Miranda et al., 2020). With both practices, windows for black soldier fly larval starvation could occur due to competition. Such delays in feeding could potentially impact black soldier fly larval microbial communities thus resulting in varied larval life history traits and subsequent production. Therefore, we took advantage of our knowledge of black soldier fly biology and feeding behavior, as well as its importance in nutrient recycling, to investigate the total associated *metabolically active* prokaryotic shifts due to host starvation and subsequent nutrient deprivation as a first step for understanding microbe-host interactive metabolism, and in contribution to the long-term goal of optimizing black soldier fly sustainable agricultural systems.

## Materials and Methods

### Colony Maintenance

Black soldier fly eggs were collected from a colony maintained in the Forensic Laboratory for Investigative Entomological Sciences (FLIES) Facility at Texas A&M University. The eggs were collected in three layers of 2 (w) by 2 (h) × 3 (l) cm corrugated cardboard blocks taped to the inside a 2 L plastic bucket 3 cm above approximately 500 g of the Gainesville diet (30% alfalfa meal, 20% corn meal, and 50% wheat bran) saturated with water (Hogsette, 1992). Cardboard was replaced daily. Cardboard containing eggs was placed in a ∼1 L deli cup and maintained in an incubator at approximately 70% RH, 27°C, and 12:12 L:D until hatch.

### Black Soldier Fly Feeding Conditions

Black soldier fly eggs, representing 66 clutches (approximately 40K eggs), were collected within an 8-h window using cardboard egg traps. Upon hatching, larvae were maintained using methods described in Sheppard et al. (2002). Larvae were fed 50 g 70% saturated Gainesville diet on the day of hatching (Sheppard et al., 2002). An additional 50 g of feed was given daily from day four to ten. On the eleventh day, 4,000 larvae were collected from the pool and partitioned equally into four containers (∼1L) (ChoiceHD brand from webstaurantstore.com), representing four technical replicates denoted as A, B, C, and D.

**FIGURE 1:**
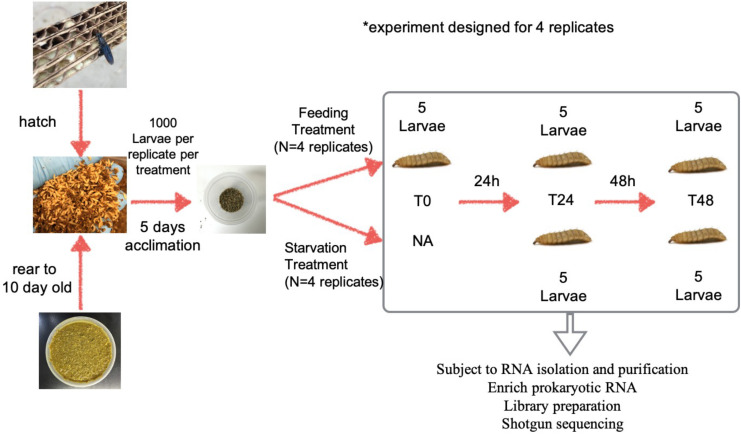
The workflow of the experiment design. Neonates hatched from the eggs were fed 70% moisture Gainesville diet until 10 days old then partitioned into 1,000 larvae per lot for an additional 5-day feeding for acclimation. On the 15th day larvae and frass were further split into two treatments of 500 larvae with equal amount of the frass each. One cup represented the control (Fed) group, in which the larvae were being fed daily (20 g, 2 h prior to the sampling, and 30 g after the sampling), until the end of the experiment. The other cup represented the Starved treatment group, in which the larvae were no longer receiving any food for the rest of the experiment. Five subsamples were taken from each replicate and subjected to RNA isolation and library preparation for shotgun sequencing.

For each replicate, larvae were fed 50 g Gainesville diet at 70% moisture daily for an additional 5 days in order to allow the larvae to acclimate to the new containers. On day 16, each replicate was split into two treatments The first treatment group represented the Control group (aka Fed group) and was fed 50 g Gainesville diet daily until the end of the experiment. The second group, representing the Starved treatment, was not fed for the remainder of the experiment. Each of the 4 replicates within the Fed group was given 20 g feed 2 h prior to larval sampling, and an additional 30 g feed after samples were taken (Figure 1).

Five sub-samples (80–100 mg each) of whole larvae (free of substrate) were taken from each replicate of the Fed and Starved groups at three time points (T0 H, T24 H, and T48 H) and placed in individual 2 ml sterile centrifuge tubes with 0.5 g glass beads and 1 mL Trizol^®^, immediately followed by manual tissue homogenization for individual RNA isolation. Whole larvae were collected in order to analyze the impact of starvation on external and internal larval microbiomes.

To have a basic understanding of the initial microbial structure in the starting feed, one subsample of the saturated 70% Gainesville diet as described above (100 mg) was taken at the beginning of the experiment. The feed sample was collected during the same time as initial larval sampling (i.e., approximately 2–3 h after being first saturated). The RNA of the feed samples was extracted using the same procedures abovementioned as all other larvae samples. Due to the single sample collection, this feed sample data was only included as a point for discussion.

### Microbial Total RNA Isolation and Purification

Manually homogenized samples were further processed with a bead beater (BioSpec) for 2 min. After the first minute of homogenization, samples were placed on ice for a 2-min incubation period to avoid overheating and subsequent RNA degradation, and then processed to the second minute of bead beating. After homogenization, samples were incubated at room temperature for 5 min, followed by centrifugation at 12,000 × *g* for 10 min at 4°C to pellet excess particulate. Following this, the supernatant in each sample was transferred to a new tube containing 0.2 mL chloroform, followed by vortexing and incubation at room temperature for 3 min. Samples were centrifuged at 12,000 × *g* for 15 min at 4°C, and resulting product was distinguished into three phases: upper, aqueous RNA phase, the middle, DNA interphase, and lower, protein and lipid organic phase.

The upper, aqueous RNA phases were transferred to a clean tube containing 0.5 mL isopropanol and incubated at room temperature for 10 min followed by a centrifugation at 12,000 × *g* for 10 min at 4°C. Supernatants were removed, and RNA pellets were washed twice using 1 mL 75% ethanol and subsequent centrifugation at 7,500 × *g* for 5 min at 4°C. The RNA pellets were air-dried, then resuspended in RNase free water. Resuspended RNA samples were pooled for each replicate, resulting in 20 RNA samples from larval samples and one RNA sample isolated from feed samples, that were stored at −20°C.

RNA was purified using the Direct-zol^TM^ RNA MiniPrep kit, following the manufacturer’s protocol. Resulting prokaryotic RNA was enriched using the MICROBEnrich^TM^ Kit, following the MICROBEnrich^TM^ Kit Protocol that captures and removes up to 90% of eukaryotic RNA, resulting prokaryotic RNA enrichment. If severe eukaryotic RNA contamination was detected, the respective samples were purified with the MICROBEnrich^TM^ Kit for a second time. The enriched RNA products were then quantified with a Qubit^®^ 2.0 Fluorometer, and ran on a gel to determine RNA quality.

The NEB Ultra RNA Library Kit (New England Biolabs) was used to convert the microbial RNA to cDNA, avoiding steps to remove rRNA, and preserving total RNA concentrations. Established cDNA libraries were multiplexed using NEBNext Oligos for Illumina to create five libraries. Resulting multiplexed libraries were submitted for shotgun whole microbial metatranscriptome sequencing using an Illumina^®^ HiSeq 2000 instrument for 2 × 101 basepair reads.

### Microbiome 16S Analyses and Functional Prediction

Raw paired-end reads from whole metatranscriptome sequences were demultiplexed, and trimmed, and adapters removed using the FasQC tool kit. Low-quality reads less than 36 nucleotides long, and low-quality nucleotide groups with a Phred + 33 score less than 30 were removed using the FasQC tool kit (Version 1.0.0). Sequences using Illumina^®^ HiSeq, followed by quality filtering, yielded 10.4 GB of sequence data (862,168 sequences) from the total 20 samples, with 101 base pair sequence length for each sample. All reads passed FastQC quality check with a mean quality score over 30.

Parallel-Meta 3.3.1 was used for bacterial identification to multiple taxonomic levels using the unassembled metagenomic shotgun sequences. Taxonomic classification and KO functional prediction were assessed by setting sequence type as “Shotgun”, and functional analysis as “Enabled”. Additionally, “format check” was disabled as instructed through personal communication with the software developer, leaving other settings as default (Su et al., 2014). Parallel-Meta utilizes high performance data mining algorithms and curated comparator databases that elucidates hundreds of thousands to millions of short reads of a metagenomic sequence into discrete microbial genomes and genes. Parallel-Meta first constructed Hidden Markov Models using all bacterial 16S rRNA sequences of SILVA (version 123), and predicted the 16S rRNA gene fragments in our shotgun samples from both the forward sequences and reversed complementary sequences by HMMER, then extracted the 16S rRNA fragments from the shotgun sequences for profiling (Eddy, 2011; Jing et al., 2017). All 16S rRNA gene sequences were aligned to the Parallel-Meta reference database by Bowtie2 for OTU picking, taxonomical annotation and phylogeny construction. The Parallel-Meta customized database integrated GreenGenes (sequence similarity on 97% level) with SILVA consensus taxonomy annotation (assigned by BLASTN with e-value < 1e-30 and similarity > 97%) (Pruesse et al., 2007), RDP (Cole et al., 2009), and Oral Core (Griffen et al., 2011) to determine microbial identification to multiple taxonomic levels. Phylogenies for reference sequences were built by FastTree (Price et al., 2009, 2010). The Parallel-Meta algorithm also rarified sequences and calculated the precise relative abundance of each organism by 16S rRNA copy number calibration using the IMG database (Markowitz et al., 2012).

Extraction of 16S rRNA yielded an average of 22,455 16S rRNA sequences per sample for taxonomic analyses, including 10 phyla, 24 classes, 40 orders, 66 families, 117 genera, 26 species, and 10691 OTUs, with the genera level providing the most informative classification. Taxa representing less than 5% in mean relative abundance across all timepoints were classified as “other”. Shannon diversity was used to measure alpha diversity, and two-tailed T-tests were performed to determine significance. Principal coordinate analysis ordination of a Bray-Curtis dissimilarity matrix was used to measure beta diversity distances, and PERMANOVA based on the Bray-Curtis dissimilarities with Bonferroni correction was used to measure significance across treatments and timepoints. To illustrate how microflora changed from the feed to larvae, we included a datum point from microbial sequencing Gainesville diet, which was collected and sequenced as a pilot study. Because we only sequenced one sample of the feed, and it cannot provide any statistical power, we only used this feed datum point for discussion to provide inferences on what microbes were originally in the feed.

Parallel Meta also implemented the PICRUSt algorithm using the KEGG database to estimate all functional gene potential within the microbiome 16S rRNA gene OTUs (Kanehisa and Goto, 2000; Langille et al., 2013). Predicted functional genes were annotated by KO (KEGG Ontology), KEGG pathways, and BRITE hierarchies (Kanehisa and Goto, 2000; Kanehisa, 2019). We determined cut-off values for a log2 fold change equaling a fold change of at least 1 in order to further rank the top 10 most significant predicted genes.

### Statistical Analyses

Additional statistical analysis was performed using RStudio (Version 0.99.903), which was built on R software (Version 3.3.1) (R Core Team, 2013). The DESeq2 package was used to detect genera with differential abundance between treatments (Love et al., 2014). The Wilcoxon-Mann-Whitney test with Benjamini-Hochberg *p*-value correction was used to determine statistical differences in the distributions of microbial taxa in the Fed and Starved groups over time. Timepoints T24 H and T48 H were treated as separate events, where raw count data from Control T24 H were tested against Starved T24 H, and Control T48 H were tested against Starved T48 H. Significance was determined with FDR adjusted *p* value (q-value) less than 0.05.

### Random Forest Feature Selection

The random forest machine learning algorithm returns an array of feature importance values of length equal to the array of input features (Liaw and Wiener, 2002). Feature importance was determined as part of the boot-strap method used for assembling random decision trees, where feature importance is greater for variables with greater predive performance. Feature ranking was determined using a bootstrap method that randomly sampled 80% of the training data from BRITE hierarchies over a default of 1000 iterations. The highest ranking values associated with BRITE hierarchies were determined over all iterations. The Wilcoxon-Mann-Whitney test with Benjamini-Hochberg *p*-value correction was used to determine statistical differences in the predicted BRITE hierarchy abundances.

## Results

### Phylum-Level Differences Between Treatments and Timepoints

**FIGURE 2:**
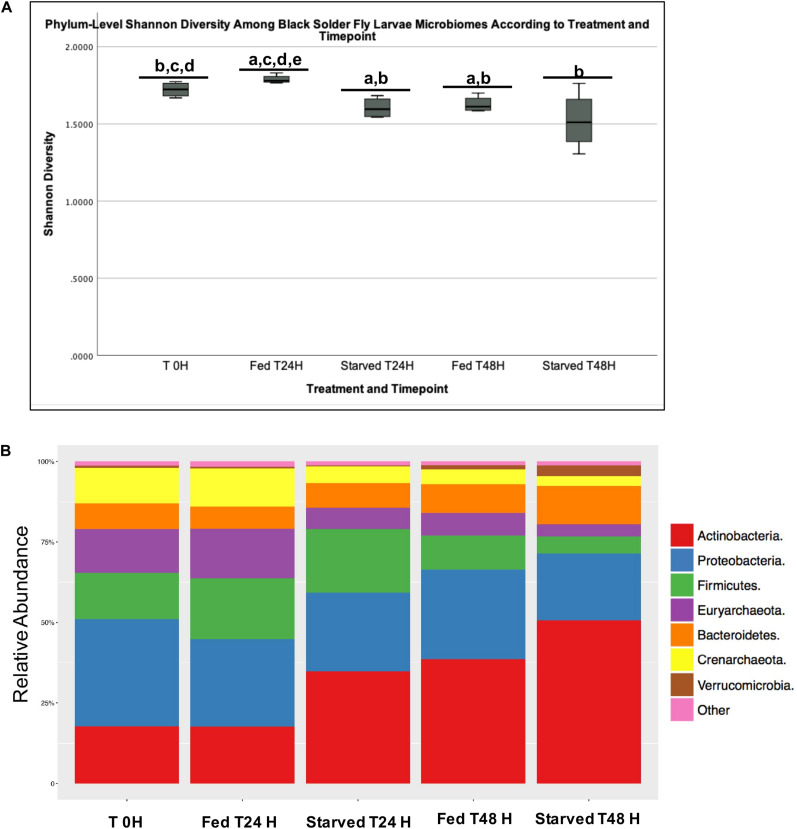
Phylum level Shannon diversity **(A)** and relative abundance **(B)** across treatments and timepoints.

The Shannon Diversity Index revealed a significantly higher diversity and a more even community overall in the Fed than in the Starved group (*p* = 0.0025) at the phylum level. The Fed group at T24 H had significantly higher Shannon diversity than the T0 H group (*p* = 0.06), as well as the group of Starved T24 H (*p* = 0.031), Fed at T48 H (*p* = 0.038), and Starved at T48 H (*p* = 0.033, Figure 2A). Shannon diversity of the Starved T24 H group was also significantly lower than the T0 H group (*p* = 0.031). Among the 11 identified phyla, six were significantly different between the Fed and Starved cohort relative abundance (q < 0.05). Actinobacteria was the most dominant phylum shared by samples from both Fed and Starved, followed by Proteobacteria, Firmicutes, Euryarchaeota, and Bacteroidetes (Figure 2B). While Actinobacteria was the most abundant phylum among all treatments, this phylum was found in significantly higher abundance in the Starved cohort (*q* < 0.001). Besides Actinobacteria, Bacteroidetes and Verrucomicrobia were also found with significantly higher abundance in the Starved cohort (*q* < 0.01 and *q* < 0.001, respectively; Table 1). On the other hand, Firmicutes, Euryarchaeota, Crenarchaeota, and Planctomycetes (found within “other” in Figure 2B) were decreased within the Starved cohort. Notably, all had decreased by 50% or more (Table 1).

**TABLE 1:** Phyla with significantly different relative abundances between Fed and Starved groups.

**Phylum**	**Mean Fed^a^**	**Mean Starved^b^**	***q*-value**
Actinobacteria	0.230	0.433	<0.001
Firmicutes	0.178	0.082	<0.001
Euryarchaeota	0.135	0.061	<0.01
Bacteroidetes	0.067	0.107	<0.01
Crenarchaeota	0.096	0.040	<0.001
Verrucomicrobia	0.005	0.027	<0.001
Planctomycetes	0.002	0.001	<0.05

*^*a*^Mean abundance among all Fed samples at T0H, T24H, and T48H.*

*^*b*^Mean abundance among all starved samples at T24H and T48H.*

### Genus-Level Differences Between Treatments and Timepoints

**FIGURE 3:**
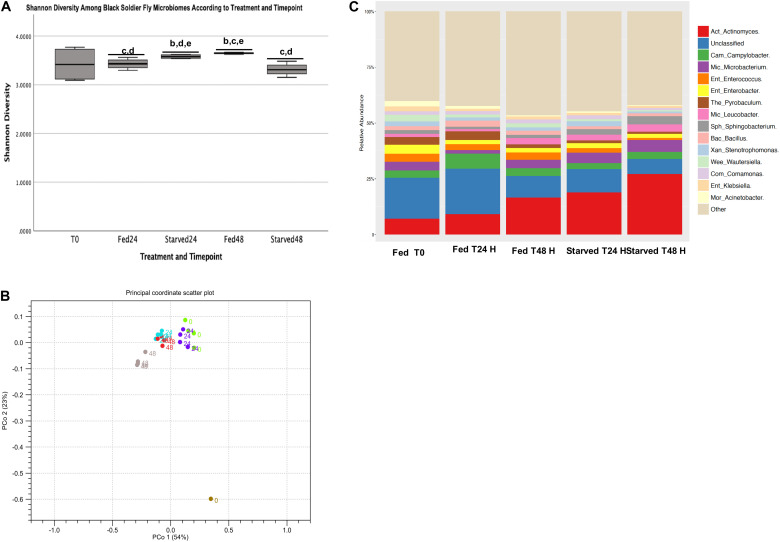
Genus level Shannon diversity and Relative Abundance. **(A)** Alpha diversity using Shannon index. **(B)** Principal coordinate plots using Bray-Curtis distances of individual fed and starved black soldier fly microbiome samples and a single feed sample. **(C)** Genus leval taxa with relative abundance greater than 5% across Fed and Starved Groups at T0, T24 H, and T48 H.

Analysis of alpha diversity using the Shannon Diversity Index showed no significant differences between the T0 H black soldier fly larval microbiomes with any other treatment or timepoint at the genus level (Figure 3A). Overall, the Fed group increased in Shannon diversity over time, while the Starved group decreased in diversity over time at the genus level. For instance, the Fed T24 H group showed significantly lower Shannon diversity at the genus level than Starved group at T24 H (*p* = 0.044) and Fed group at T48 H (*p* = 0.008). The Starved group at T24 H was significantly lower in alpha diversity compared to the Fed group at T48 H group, but was significantly higher than the Starved T48 H group. Bray Curtis Distances were computed at the genus level and showed individual samples clustering together according to treatment and timepoint (Figure 3B). Samples from the Fed group at T24 H clustered closest to the T0 samples, while the T24 H Starved group clustered closest to the T48 H Fed group. Individual samples from the Starved group at T48 H clustered with each other, but further away from the other samples. The single Feed sample showed greatest separation from the larval samples (Figure 3B). But, analysis of clustering across and between cohorts using PERMANOVA with inclusion of Bonferroni adjustment was not statistically significant.

Taxa with relative abundance greater than 5% across all Fed and Starved groups and timepoints are shown in Figure 3C (individual relative abundance in each sample across treatments and timepoints are shown in Supplementary Figure 1 and Supplementary Table 1). Of these, *Enterobacter, Stenotrophomonas, Wautersiella, Klebsiella* and *Comamonas* were not statistically different between Starved and Fed groups at either T24 H or T48 H timepoints. These genera also had mean relative abundances of 2.0, 1.5, 1.2, and 1.5%, respectively, across the timepoints.

#### Statistically Significant Differences in Black Soldier Fly Microbiome Relative Abundances at Either the Twenty-Four or Forty-Eight Hour Timepoints

A total of 56 identified genera had significantly (*q* < 0.05) different abundances between treatments at only the T24 H, and 39 genera at only the T48 H timepoint (Supplementary Tables 1, 2). Among the most abundant taxa (greater than 5% across treatments and timepoints), *Campylobacter* relative abundance doubled between T0 and T24 H in the Fed samples, but then decreased to initial abundance, comparable also to the Starved samples (Figure 3C). However, there was a statistically significant difference in *Campylobacter* relative abundance between Fed and Starved at the 24 H timepoint (6.8% versus 3.2%, *p* = 0.0006). *Pyrobaculum* relative abundance increased from 3.3% at T0 to 3.9% at T24 H in relative abundance in the Fed group and was statistically higher in relative abundance than the Starved group (*p* < 0.001), which decreased from 1.3% at the 24 H timepoint to 0.95% at the 48 H timepoint in the Starved Group (Figure 3C). *Bacillus* relative abundance in the Fed group had an initial increase between T0 and T 24 H (from 1.8 to 2.7%) that was statistically higher than the Starved group (*p* = 0.0012), but then decreased to 1.7% at T 48 H. *Bacillus* relative abundance in the Starved group remained relatively constant (1.7 and 1.3%, respectively). Finally, *Acinetobacter* decreased across all timepoints in both treatments from 2.4 at T0 to 1.2 and 0.55%, in the Fed group and to 0.7 and 0.3%in the Starved group at T24 H and T48 H), but was statistically higher in the Fed group than in the Starved group at T24 H (*p* < 0.001). Unclassified taxa accounted for statistically significant higher (10.5–22.3%, *p* < 0.001) relative abundance in the Fed group, with only 8.3–11.1% in the Starved group across timepoints (Figure 3C). While there were statistically significant differences between treatments at the T48 H timepoint (Supplementary Table 2), taxa represented relative abundances less than 5% across all timepoints.

### Statistically Significant Differences in Black Soldier Fly Microbiome Relative Abundances at Both Twenty-Four and Forty-Eight Hour Timepoint

**FIGURE 4:**
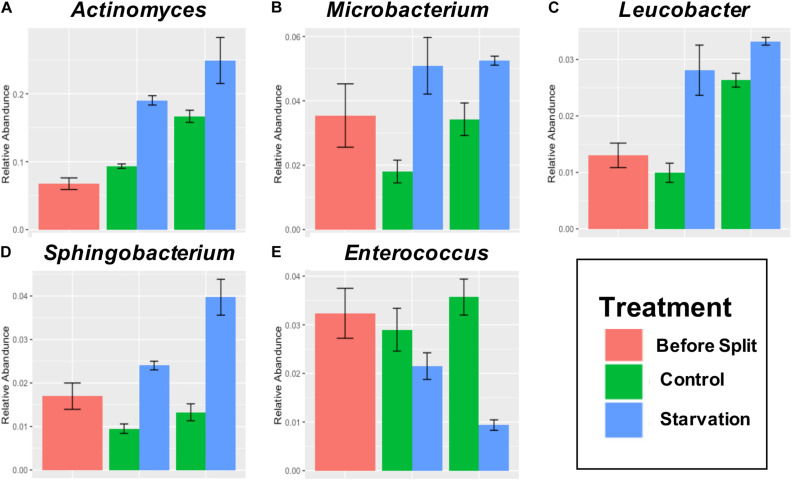
Mean relative abundance greater than 5% and significantly different (*q* < 0.05) abundance at both T24 H and T48 H for Genera *Actinomyces* **(A)**, *Microbacterium* **(B)**, *Leucobacter* **(C)**, *Sphingobacterium* **(D)**, and *Enterococcus* **(E)**. T0 is the initial timepoint prior to division into Fed and Starved treatments.

Five genera with a minimum abundance of 5% at any treatment or timepoint were identified as significantly different at both the T24 H and T48 H (Figures 3, 4). *Actinomyces* was identified as the most abundant genus with an average abundance of 15.8% across all samples. *Actinomyces* increased abundance over time in larval samples, no matter the treatment (Figure 4A). Additionally, *Actinomyces* was statistically significantly higher in the Starved treatment (19%, *p* < 0.001, Figure 4A) than the Fed (9.2%) at the 24H timepoint and, and also at the T48 H timepoint (27% Starved versus 17% fed, *p* < 0.001). *Microbacterium* was also significantly higher in the Starved group at T24 H (4.6% in Starved versus 1.5% in Fed, *p* = 0.0005) and at T48 H (5.3% in the Starved versus 3.7% in Fed, *p* < 0.001, Figure 4B). *Leucobacter* relative abundance was also statistically significantly different between the treatments at the T24 H and T48 H timepoints with higher relative abundance in the Starved group. *Leucobacter* relative abundance was 0.98% in Fed versus 2.6% in Starved at T24 H (*p* = 0.0008), and was 2.8% in Fed versus 3.3% in Starved at T48 H (*p* < 0.001, Figure 4C). *Sphingobacterium* relative abundance was also significantly higher in the Starved group at both timepoints (*p* = 0.0003 at T24 H and *p* < 0.0001 at T48 H) where it increased from 2.5% at T 24 H to 3.7% at T 48 H versus 1.1 and 1.5% in the Fed group at T24 H and T 48 H, respectively (Figure 4D). *Enterococcus* remained relatively constant across the Fed group from T24 H to T 48 H (2.6, and 3.3%, respectively), but was significantly higher (*p* = 0.0084 and *p* < 0.001 at T24 H and T48 H, respectively, Figure 4E) than the Starved group that decreased to 2.0% at T24 H and further decreased to 0.9% at T48 H.

### Functional Prediction

The functions of black soldier fly larval microbiomes were predicted using PICRUSt analysis. With this, 16S rRNA gene sequencing data were categorized into 6,709 KEGG functional pathways (Supplementary Table 3). Pathways present in <10% of all samples were removed, leaving 5,905 KEGG pathways for comparison. The DESeq2 package identified 4,401 predicted gene abundances that were statistically different (*q* < 0.05) between Starved and Fed groups (Supplementary Table 4). We focused on predicted genes with log2 fold change cut-offs yielding fold changes of at least 1, and identified the 10 predicted genes down regulated and 10 predicted genes upregulated (Table 2 and Supplementary Table 4) in the Starved group. Of those upregulated, three showed predicted function in carbohydrate metabolism, two were predicted to be involved in metabolism of other amino acids, and three were predicted to be involved in metabolism of cofactors, terpenoids, or methane, respectively. Many of these predicted genes are also known to be involved in microbial metabolism in diverse environments. The remaining two of the ten significantly upregulated had predicted functions in environmental information processing and signal transduction (Table 2).

**TABLE 2:** The top 10 significantly upregulated and downregulated predicted genes in the Starved treatment with log2 fold changes greater than 1 (means), along with their KEGG annotations (listed in order of q-value followed by log2 fold change).

**KEGG orthology**	**log2 fold change**	***q*-value**	**Gene description**	**KEGG BRITE functional classifications**
K14448	1.92	3.93E-31	(2*S*)-methylsuccinyl-CoA dehydrogenase	Carbohydrate metabolism; Glyoxylate and dicarboxylate metabolism; Microbial metabolism in diverse environments
K03851	1.95	2.33E-29	taurine-pyruvate aminotransferase	Metabolism of other amino acids; Taurine and hypotaurine metabolism
K04036	1.88	2.33E-29	divinyl protochlorophyllide a 8-vinyl-reductase	Metabolism of cofactors and vitamins; Porphyrin and chlorophyll metabolism
K14451	2.03	2.73E-29	(3*S*)-malyl-CoA thioesterase	Carbohydrate metabolism; Glyoxylate and dicarboxylate metabolism; Microbial metabolism in diverse environments
K09847	2.01	2.56E-27	spheroidene monooxygenase	Metabolism of terpenoids and polyketides; Carotenoid biosynthesis
K08927	1.62	5.14E-24	light-harvesting complex 1 beta chain	Environmental Information Processing; Signal transduction
K14083	1.32	6.37E-26	trimethylamine—corrinoid protein Co-methyltransferase	Methane metabolism; Energy metabolism; Microbial metabolism in diverse environments/methanogenesis
K05915	1.78	7.62E-26	(hydroxymethyl)phosphonate/2-amino-1-hydroxyethylphosphonate	Metabolism of other amino acids; Phosphonate and phosphinate metabolism
K14449	1.60	3.08E-25	2-methylfumaryl-CoA hydratase	Carbohydrate metabolism; Glyoxylate and dicarboxylate metabolism; Microbial metabolism in diverse environments
K07772	−1.35	9.39E-24	two-component system, OmpR family, torCAD operon response regulator TorR	Environmental Information Processing; Signal transduction
K03346	−1.00	2.33E-13	Replication initiation and membrane attachment protein	Genetic information processing; DNA replication proteins
K12269	−2.43	2.11E-11	Accessory secretory protein Asp2	Signaling and cellular processes; Secretion system
K12270	−2.32	3.16E-11	Accessory secretory protein Asp3	Signaling and cellular processes; Secretion system
K07683	−1.65	3.27E-11	Two-component system, NarL family, sensor histidine kinase NreB	Environmental Information Processing; Two-component system, NarL family, sensor histidine kinase NreB
K02828	−1.30	3.88E-11	Cytochrome aa3-600 menaquinol oxidase subunit III	Energy metabolism; Cytochrome aa3-600 menaquinol oxidase subunit III
K03697	−1.04	4.97E-11	ATP-dependent Clp protease ATP-binding subunit ClpE	Genetic information processing; Chaperones and folding catalysts
K10984	−1.03	5.08E-11	Galactosamine PTS system EIIB component	Carbohydrate metabolism; Galactose metabolism
K02245	−1.02	5.98E-11	Competence protein ComGC	Signaling and cellular processes; Type II secretion system
K02240	−1.02	5.98E-11	Competence protein ComFA	Signaling and cellular processes; Type II secretion system
K07570	−1.01	6.44E-11	General stress protein 13	Genetic information processing; Translation

Three of the ten predicted genes that were significantly downregulated in KO abundance in the Starved group were placed within the genetic information processing category and included genes predicted to be involved in DNA replication, chaperones, and general stress response (Table 2). Four additional downregulated genes were predicted to be involved in signaling and cellular processes as part of secretion systems. The remaining three significantly downregulated were predicted to be involved in environmental information processing, energy metabolism, and carbohydrate metabolism, respectively.

**FIGURE 5:**
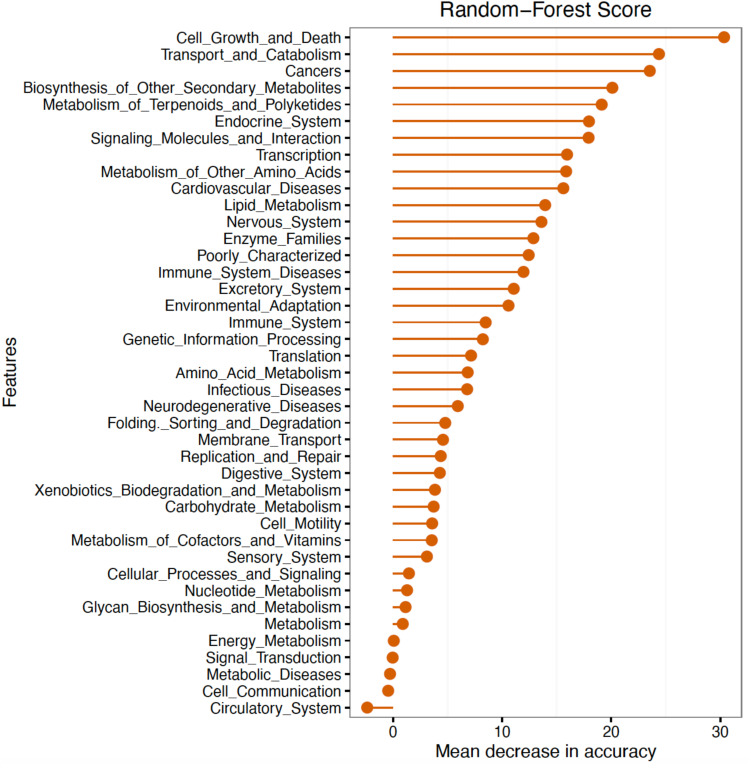
Random forest classification of BRITE hierarchies as most important predictors of Fed versus Starved Groups.

Further analyses of mean abundance of predicted genes classified 16S rRNA sequences into 31 BRITE hierarchies. The random forest algorithm was applied to determine whether these predicted BRITE hierarchies could differentiate between Fed and Starved groups. The mean decrease in accuracy of BRITE hierarchy attributes showed the most relevant descriptors were associated with cell growth and death, transport and catabolism, cancers/human diseases, biosynthesis of other secondary metabolites, and metabolism of terpenoids and polyketides (Figure 5). BRITE hierarchy features that were statistically significantly predicted to be most associated with Starved or Fed groups are summarized in Table 3.

**TABLE 3:** BRITE hierarchy features statistically significantly associated with Starved or Fed groups using the Wilcoxon Rank Sum Test with Benjamini Hochberg (BH) *p*-value correction.

**BRITE hierarchy**	**Mean Fed**	**Mean Starved**	**Standard Deviation Fed**	**Standard deviation Starved**	**Wilcoxon-BH**
Cell growth and death	4.38E-03	4.84E-03	3.49E-04	1.08E-04	6.16E-05
Biosynthesis of other secondary metabolites	8.65E-03	9.35E-03	1.91E-04	3.67E-04	6.16E-05
Transport and catabolism	2.87E-03	3.42E-03	2.36E-04	1.98E-04	6.16E-05
Endocrine system	3.44E-03	4.14E-03	3.61E-04	3.22E-04	6.16E-05
Signaling molecules and interaction	1.66E-03	1.95E-03	2.01E-04	1.11E-04	8.82E-05
Cancers	1.22E-03	1.47E-03	1.37E-04	7.99E-05	8.82E-05
Lipid metabolism	3.04E-02	3.25E-02	1.71E-03	1.65E-03	2.81E-04
Metabolism of other amino acids	1.68E-02	1.76E-02	8.10E-04	3.37E-04	2.82E-04
Environmental adaptation	1.24E-03	1.44E-03	1.32E-04	1.38E-04	3.31E-04
Transcription	2.68E-02	2.57E-02	1.16E-03	3.61E-04	3.92E-04
Poorly characterized	5.51E-02	5.18E-02	3.13E-03	1.14E-03	4.66E-04
Metabolism of terpenoids and polyketides	1.87E-02	1.94E-02	5.29E-04	2.01E-04	5.57E-04
Translation	5.48E-02	5.02E-02	4.69E-03	2.82E-03	3.28E-03
Genetic information processing	2.67E-02	2.50E-02	1.64E-03	5.33E-04	3.83E-03
Amino acid metabolism	9.99E-02	1.02E-01	2.14E-03	1.31E-03	5.58E-03
Enzyme families	1.93E-02	1.89E-02	7.24E-04	3.36E-04	5.83E-03
Neurodegenerative diseases	2.25E-03	2.42E-03	2.55E-04	1.92E-04	6.80E-03
Infectious diseases	4.04E-03	3.75E-03	4.21E-04	2.74E-04	1.95E-02
Glycan biosynthesis and metabolism	1.81E-02	1.92E-02	1.12E-03	1.27E-03	2.67E-02
Xenobiotics biodegradation and metabolism	2.64E-02	2.78E-02	2.20E-03	1.70E-03	2.90E-02
Metabolism of cofactors and vitamins	4.10E-02	4.15E-02	1.41E-03	8.36E-04	2.90E-02

## Discussion

Our data demonstrate that starvation impacts microbial community structure in the larvae. Such impacts were demonstrated across taxonomic scale (i.e., phylum and genus) and predicted gene function. This study represents not only the first to demonstrate such impacts on black soldier fly larvae but is also among only a few on insects in general.

As discussed below, research on starvation has primarily focused on vertebrates. Understanding such influences across taxa is critical for deciphering the mechanisms regulating these responses as a means to develop strategies for combating health-related benefits. From an industrial perspective of insect farming, once described, such processes could be manipulated to enhance production of the targeted insect, such as the black soldier fly, or the inverse- prolong development as a means to enhance waste conversion. Both outcomes could be massively important for stabilizing the industry as they could lead to greater efficiency in production or maintenance of a larval ‘bank’ that can be used when organic streams surpass facility needs.

Starvation could impact final product output. Inabilities to feed black soldier flies at a consistent rate throughout larval development could impact waste conversion and growth patterns. Previous reviews discuss the impact of diet nutrition on the protein and fat composition of harvested black soldier fly larvae (see reviews by Barragan-Fonseca et al. (2017), Gold et al. (2018), and Surendra et al. (2020). And, of equal importance, and discussed in the previous reviews cited, nutrient composition of resulting larvae could also be impacted. Given the black soldier fly larvae are produced for their protein and fat, failure to optimize the process could result in product not meeting regulatory or industrial standards.

While many studies have focused on the impact of adding nutrients into a system on associated gut microbiome structure, a literature review revealed only limited investigations of bacterial community shifts during host starvation (Xia et al., 2014; Davis, 2016; Seitz et al., 2019; Cornide-Petronio et al., 2020; Golonka et al., 2020; Jing et al., 2020; Schroder et al., 2020). In fact, little is known about the impact of fasting on the microbiome of invertebrates. Tinker and Ottesen (2016) demonstrated stability in the microbiome of the gut of the American Cockroach, *Periplaneta americana* (Blattodea: Blattidae) when starved (Tinker and Ottesen, 2016). Interestingly, their results contrast with those demonstrated in the current study; however, it is hypothesized that such differences could be due to life-history differences with the American roach having a longer life-span (e.g., 12 months) with adults feeding, and the black soldier fly adult have a comparatively short life-span with little reliance on adult nutrition (Rau, 1940; Tomberlin et al., 2002; Bertino-Grimaldi et al., 2013).

Clearly, in situations where nutrients are deficient, the host can experience starvation depending upon the circumstances. And, it can be expected that in such situations, similar downstream effects are observed for the commensal microbes. This impact has been well-documented for vertebrate models. For instance, Costello et al. (2010) demonstrated an abundance of Bacteroidetes during fasting that shifted toward a post-prandial abundance of Firmicutes in the Burmese python. Similarly, Crawford et al. showed similar results when starving mice for 24 H (Crawford et al., 2009). Our results demonstrate similar patterns indicating mechanisms of microbiome responses to fasting (i.e., starvation) are potentially convergent across taxa. Such mechanisms, once deciphered, could be put into practice to enhance, or at minimum stabilize, black soldier fly production associated nutrient content.

As mentioned initially in the discussion, achieving such advancements are critical for optimizing black soldier fly farming for more accurate projections in production. For example, one possible approach to optimize production is to keep black soldier fly larvae hungry, by maintaining a microbiome that is associated with host hunger (i.e., predicted function). In such a system, “hunger-related” microbes (i.e., *Enterococcus* species within the current study) found internally or externally, might serve as signaling agents for hunger induction in the larval host. This hunger trigger would presumably promote increased feeding, and ultimately waste to protein conversion, meeting farmers’ goals for higher biomass per capita; especially in the black soldier fly larvae production industry. Similarly, microbial community members may be identified that provide added nutritional value and utility in waste conversion.

Starvation could also reduce microbial community structure, as seen in our study (Fed v Starved, Figures 2, 3) allowing for easier manipulations for greater production. Decreased microbial diversity, which tends to be associated with larger populations of the same species (i.e., microbes in gut of insect), often result in individual species having more energy and resources and a higher capacity for host manipulation, because fewer resources are spent on competition. In this instance, bacterial cross-feeding, wherein one species of bacteria provides nutrients for another species, may also be occurring (Smith et al., 2019). This concept agrees with other studies that showed bacterial metabolites and cross-feeding can influence host satiety pathways, and have extensive effects on appetite and feeding behavior (Fetissov, 2017; Yang and Tomberlin, 2020).

Starving larvae did not impact the presence of some key microbial phyla. Actinobacteria was the most dominant metabolically active phylum shared by samples from both Fed and Starved groups, but was found in higher abundance in the Starved cohort. Additionally, Bacteroidetes and Verrucomicrobia were also found with higher abundance in the Starved cohort. On the other hand, Planctomycetes, Firmicutes, Euryarchaeota, and Crenarchaeota, showed higher relative abundance among the Fed cohort. Planctomycetes were previously considered to be only found in marine environments, however recent data has shown members of the phylum to be associated with insect and human microbiomes (Franzini et al., 2016).

Euryarchaeota and Crenarchaeota are archaeal phyla. Archaea represent a large proportion of the Earth’s ecosystem, with the capability of residing in diverse and extreme environments (Eme and Doolittle, 2015). Many archaea are substantial components of complex microbial communities, including in plant and animal microbiomes (Lurie-Weinberger and Gophna, 2015; Moissl-Eichinger et al., 2018). And, archaeal sequences have been identified in association with microbiomes of black soldier flies raised on low bioburden diets, though at low relative abundances, suggesting that these phyla are normal black soldier fly residents, aiding in digestion (Klammsteiner et al., 2020). Our shotgun data showed highest archaeal resolution at the order level, with four archaeal orders, including Halobacteriales, Methanobacteriales, Methanomicrobiales, and Thermoproteales (Supplementary Table 1). All of the orders showed a statistically significant reduction in relative abundance in the Fed group from T24 H to T48 H with even lower relative abundance in the Starved group. There was a statistically significantly lower relative abundance in the Starved group than Fed group at T24 H for all detected archaeal orders, however there was no statistically significant difference between the two groups at T48 H (Supplementary Table 1, and data not shown). With the exception of Methanobacteriales, the mean relative abundances ranged from 0.13 to 3.6% across all treatments and timepoints, and were at very low relative abundances or absent from the single feed sample (Supplementary Table 1). Methanobacteriales had a mean relative abundance of 14.7% at T0 and 14.6% at T24 H in the Fed group, decreasing to 5.9% at T48 H. The Starved group had a mean relative abundance of 6.4% Methanobacteriales, decreasing to 4.2% at T48 H. The single feed sample showed a 6.7% relative abundance of Methanobacteriales. Methanomicrobiales and Methanobacteriales are well defined within the Euryarchaeota phylum, and are hydrogenotrophic organisms that carry out methanogenesis. These archaea are capable of entering into syntrophic relationships with other gut microbes, and likely stimulate symbiotic digestion of plant material, and the process of methanotrophy used by other consortial microbes for sources of carbon and energy (Mitsumori et al., 2002; Reuss et al., 2015). Members of Methanobacteriales also have a limited range of catabolic substrates, utilizing H_2_, CO_2_, CO, formate, and C_1_-metyhlated compounds (Bonin and Boone, 2006). Although methane emission was not measured in this study, the high relative abundance of methanogenic archaea in the Fed group at T0 and T24 H is interesting, especially with regards to several reports of low methane emission rates by black soldier fly larvae (Mertenat et al., 2019; Pang et al., 2020; Parodi et al., 2020). Indeed, differences in methanogen abundance would affect methane production, but it remains also possible that differences in microbial composition and functioning that result in perturbation of hydrogen metabolism or accumulation may also impact methanogenesis and methane emissions (Sugimoto et al., 1998; Sollinger et al., 2018; Aguilar-Marin et al., 2020). Notwithstanding, the higher relative abundance of Methanomicrobiales in the black soldier fly larval microbiomes within the early timepoints of the Fed group could be an indication of the reduced gut environment, as well as conditions of the external (i.e., fresh substrate with very little frass) and consumed substrate (i.e., within the gut), including mixed alcohol and other products resulting from digestion and fermentation of plant polymers (Lurie-Weinberger and Gophna, 2015; Moissl-Eichinger et al., 2018). The presence of metabolically active methanogens in the feed sample could be due to the addition of water for saturation prior to sampling that might allow for fermentation and a more reduced environment to occur, and products that could be conducive for methanogen enrichment. However, a more meaningful assessment of saturated Gainesville diet with a sample size conducive for increased power would be necessary in order to further, or confirm this hypothesis. Halobacteriales and Thermoproteales are both fron the Crenarchaeota phylum and have been identified in human and insect microbiomes (Borrel et al., 2014; Gaci et al., 2014; Douglas, 2015; Prasad et al., 2018; Kim et al., 2020).

Enhancing the taxonomic resolution to genus level revealed overall microbial structure was impacted by starvation. Among genera with at least 5% mean relative abundance across all treatments and timepoints, *Actinomyces*, *Microbacterium*, *Enterococcus*, *Sphingobacterium*, and *Leucobacter* showed significantly different relative abundance at both time points, with higher relative abundances in the Starved group. This suggests that starvation resulted in some microbial members becoming dominant and outcompeting those not able to tolerate starvation stress. These dominating bacteria may also aid in host adaptation to starvation. For instance, *Actinomyces* is known to produce enzymes for degradation of chitin and plant material, and is also known to produce antibiotics against pathogenic fungus (Sharma, 2014). These microbial characteristics could help the host survive during prolonged nutrient deprivation. Taxa were identified that were found in both Starved and Fed groups, as well as those significantly associated individually with either group. Taxa identified in both groups, but not found in the feed, could constitute members of a core microbiome, though their specific role in host biology needs further investigation. And, as the feed sample was only a *N* = 1, additional studies using multiple replicates would be necessary. Indeed, a recent study by Wynants et al. showed that black soldier fly larval microbiomes and substrate-associated microbes differed substantially, but also that there were some shared taxa between larvae, despite differences in rearing conditions (Wynants et al., 2019). Many of the genera in our study were also found in their study. Further, another study found that *Actinomyces* and *Enterococcus* were among those making up the core microbiome (Klammsteiner et al., 2020).

Our microbiome data differ from those generated in other black soldier fly studies. For instance, Zheng et al. (2013) surveyed standard grain diet fed 7-d-old black soldier fly larvae associated symbionts, and found, that Bacteroidetes (54.4%) was the most abundant phylum, followed by Firmicutes and Proteobacteria. Jeon et al. (2011) surveyed three groups of 8-d-old black soldier fly larval gut microflora fed with restaurant waste, cooked rice, and calf forage, and found that the microbial community structures were directly influenced by the intake feed. For example, those fed restaurant waste showed Bacteroidetes (67.4%) and Proteobacteria (18.9%) as predominant phyla, whereas Proteobacteria (54%) and Firmicutes (42.3%) were dominant when black soldier fly larvae fed on cooked rice. Larvae who fed on calf forage had a more evenly distributed community with Proteobacteria (31.1%), Actinobacteria (24.6%), Firmicutes (23.5%), and Bacteroidetes (20.5%) (Jeon et al., 2011). In both of these previous studies, Bacteroidetes was found to be the most abundant. In our studies, black soldier fly larvae fed Gainesville diet at T0 H (11-d-old), had gut microbiomes predominated by Proteobacteria (31.4%), followed by Euryarchaeota (16.9%) and Actinobacteria (16.6%). These differences underscore the importance of diet considerations in selective pressure for influence on resident gut microbiomes. Other contributors, such as larval age, rearing environment, host genetics, and early microbial exposure may also account for some differences. It is also worth mentioning that these previous studies used 16S rRNA targeted amplicon pyrosequencing techniques, while our study used a total RNA-based shotgun sequencing technology, which provides greater resolution and deeper analysis for strain diversity, and profiled the metabolically active community, potentially explaining some of the community profile differences. It is also likely, as has been shown in other studies, that larval density, diet, temperature, and other abiotic and biotic factors in our study may be driving microbial richness and evenness (Bruno et al., 2019; Wynants et al., 2019; Klammsteiner et al., 2020). Furthermore, differences in microbial richness, evenness, and relative abundance could also be accounted for in differing locations of the midgut. We sampled the internal and external microbiome of black soldier fly larvae, but recent studies have shown that structural, biological and functional differences exist in the midgut that drive microbial diversity (Bruno et al., 2019; Bonelli et al., 2020).

Additionally, bacteria are likely to show a group stress response under starvation conditions (Li and Tian, 2012). In fact, a growing body of knowledge has shown that quorum-sensing molecules are responsible for inter-kingdom communication (Hughes and Sperandio, 2008). As such, microbes possibly, through mechanisms of modulated host or microbe genes, hijacking host signals, or production of microbial chemical signals, induce host dysphoria to induce host hunger and consequently, nutrient intake. Therefore, besides the information of bacterial structure, information of functional potential according to host nutritional status is also important. PICRUSt utilizes characterized bacterial genomes and phylogenetic relationships to predict the functional genomes of other bacteria within the constructed phylogeny based on 16S marker gene data. PICRUSt has been validated by the Human Microbiome Project and has been utilized for functional prediction across a diverse array of microbiome datasets (Fazlollahi et al., 2018; Ferreira et al., 2018; Reyes-Sosa et al., 2018; Wilkinson et al., 2018; Miao et al., 2020). But, it is important to note that functional prediction is not the same as true function. Indeed PICRUSt infers functional potential based on 16S rRNA sequences, and as such may over predict the number of pathways with significant difference over time and treatment compared to true metatranscriptome datasets because predictions are based on microbial community structure (Wilkinson et al., 2018). It will be important and informative to determine the level of accuracy of PICRUST in comparison to true functionality, versus over or under inflated predictions of pathways between genes.

Starvation impacted predicted microbial function associated with black soldier fly larvae. For instance, our data showed differences in black soldier fly larvae microbiome functional potential between microbiomes of Starved or Fed black soldier fly larvae. Three of the top 10 predicted downregulated KO abundances were associated with genetic processing. Predicted genes included those for a replication initiation and membrane attachment protein, an ATP-dependent Clp protease ATP-binding subunit ClpE that is a chaperone or folding catalyst, and for a general stress protein involved in translation (Table 2). A predicted gene encoding a galactosamine PTS system EIIB component was also predicted to be downregulated in the Starved group. These genes typically function in carbohydrate metabolism and have beta galactosidase activity (UniProt Consortium, 2018). The *qoxC* gene was also predicted to be downregulated. This gene is involved in energy metabolism (UniProt Consortium, 2018). Four of the top 10 significantly lower KO abundances were predicted genes involved in signaling and cellular processes and were part of secretion systems. And finally, one predicted gene with significantly lower KO abundance in the Starved group was involved in Environmental Information Processing and was part of a two-component system, NarL family, sensor histidine kinase NreB involved with dissimilatory nitrate reduction. Overall, predicted downregulation in genetic information processing and metabolism in the Starved cohort, indicates not only a microbial structure shift, but also that some bacteria in the starved host might have entered a dormant stage in response to the lack of nutrients, and have also altered metabolic pathways in response to changing carbon and energy sources (Tables 2, 3). Furthermore, downregulation in secretion systems and energy metabolism suggests an means to conserve energy.

On the other hand, three of the 10 significantly predicted upregulated genes were those for glyoxylate and dicarboxylate metabolism. These genes are involved in carbon fixation pathways in prokaryotes that leads to the biosynthesis of carbohydrates from fatty acids or two-carbon precursors. These genes are also active during microbial metabolism in diverse environments (UniProt Consortium, 2018). Other predicted upregulated genes include those for carotenoid synthesis, methane metabolism, a gene encoding a light-harvesting complex 1 beta chain involved in signal transduction and a gene involved in metabolism of cofactors and vitamins. Finally, two predicted upregulated genes included those for taurine and hypotaurine metabolism and phosphonate and phosphinate metabolism, respectively. These as well as significantly different BRITE hierarchies (Tables 2, 3) suggests shifts in the metabolically active microbial community structure to those organisms utilizing these functions, but also suggests a shift to a more diverse metabolism and utilization of carbon sources as well as to functional stress responses. Therefore, predicted functional responses may reflect selective pressures applied by the host but may also denote a competitive survival strategy for these bacteria to adapt to different environmental conditions. Whether predictive gene regulation shown here are demonstrative of responses to specific host signals in this context is unclear, and will be an interesting avenue for further investigation.

Finally, our taxonomic resolution was able to identify specific genera; however, only 17.7% sequences were identified on the species level, indicating these sequences belonged to poorly characterized microbial lineages associated with black soldier fly larvae. Species identification within our samples will be necessary to achieve any targeted manipulation as well as to determine probiotic versus pathogenic potential. For instance, though many *Weissella* strains have both pre- and probiotic properties, some are opportunistic pathogens to humans, and *W. ceti* is pathogenic to farmed rainbow trout (*Oncorhynchus mykiss*) (Figueiredo et al., 2012). Further investigations are required to determine if such a community structure containing possible pathogenic bacteria in black soldier fly larvae would cause animal infections. In addition, *Campylobacter* was found in both Fed and Starved larvae (Supplementary Table 1). Some *Campylobacter* ssp. are human pathogens causing diarrhea, cramping, abdominal pain, and fever in those infected, and is the most common cause of diarrheal illness in the United States (CDC, 2014). *Campylobacter* organisms are present in livestock such as chicken flocks, and can be transmitted through common water sources and contact with infected feces, and most *Campylobacter* can be easily killed through proper handling (CDC, 2014). Recently, *Campylobacter* was detected in abundance greater than 1.5% in larvae from two large-scale rearing facilities, but whether these represent commensals, transient microbes, or pathogenic organisms remain unknown (Wynants et al., 2019). Thus these findings merit more in-depth study.

In conclusion, our data revealed that starvation of the black soldier fly larval host led to a dramatic shift in the metabolically active microbiomes. Although the biological meaning of such a shift remains undetermined, investigating the functions of those specific taxa associated with nutrient rich or replete states are valuable for elucidating inter-kingdom communication and contribution of microbes in waste conversion and nutrient uptake. This increased knowledge will allow utility for designing methods and strategies for improved black soldier fly bioconversion efficiency through microbial manipulation to evolve into a solution of increasing protein production and waste reduction.

## Data Availability Statement

The datasets presented in this study can be found in online repositories. The names of the repository/repositories and accession number(s) can be found in the article/Supplementary Material.

## Author Contributions

FY and HJ designed and performed the experiment, analyzed the data, and wrote the draft. JT designed the experiment and wrote the draft. HJ organized the communication. All authors contributed to the article and approved the submitted version.

## Conflict of Interest

The authors declare that the research was conducted in the absence of any commercial or financial relationships that could be construed as a potential conflict of interest.

## Supplementary Material

Supplementary Figure 1 Genus-level relative abundances of individual samples across treatments and timepoints.

Supplementary Table 1 Relative abundance of taxa across samples.

Supplementary Table 2 DESeq2 results of genera with significantly different abundances between Starved and Fed larvae at T24 H and T48 H. A total of 82 genera had significantly (*q* < 0.05) different abundance between treatments within the T24 H time point, and 64 genera had significant differences in abundances between treatments within the T48 H time point. Highlighted cells indicate the top six genera that were statistically significantly different in abundance across both time points.

Supplementary Table 3 Predicted KEGG Orthology based on hylogenetic Investigation of Communities by Reconstruction of Unobserved States (PICRUSt).

Supplementary Table 4 Significantly different Predicted Gene Abundances Based on DeSEQ2 Analyses.
