# Supplementary figures and images for: Starvation Alters Gut Microbiome in Black Soldier Fly (Diptera: Stratiomyidae) Larvae
Source: Front Microbiol. 2021 Feb 16;12:601253. doi: 10.3389/fmicb.2021.601253 (PMC7921171; doi:10.3389/fmicb.2021.601253)

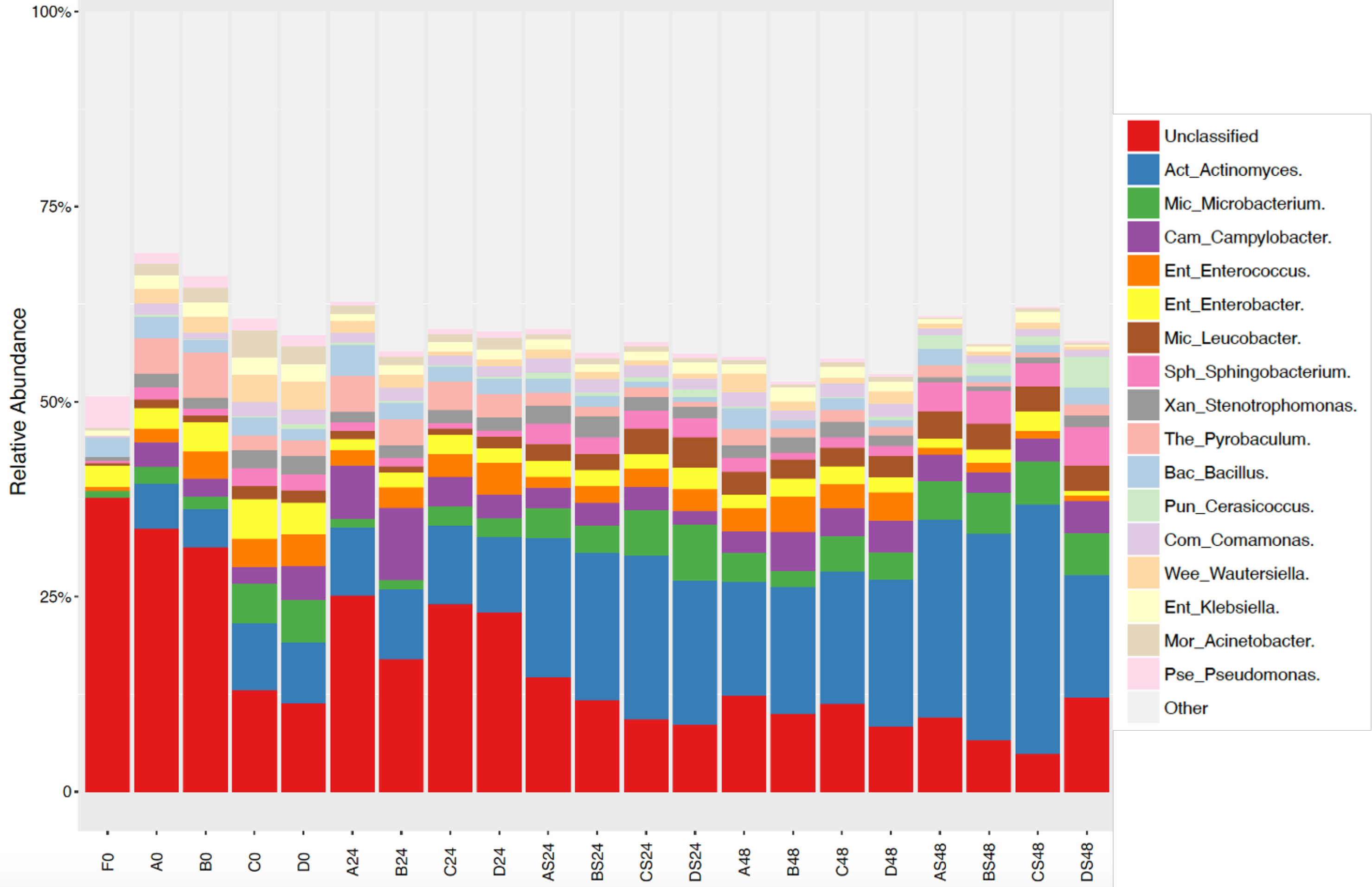

Supplement: Supplementary Figure 1 — Genus-level relative abundances of individual samples across treatments and timepoints. [file Image_1.tif]
