# Supplementary material for: Starvation Alters Gut Microbiome in Black Soldier Fly (Diptera: Stratiomyidae) Larvae
Source: Front Microbiol. 2021 Feb 16;12:601253. doi: 10.3389/fmicb.2021.601253 (PMC7921171; doi:10.3389/fmicb.2021.601253)
Supplement: Supplementary Table 2 — DESeq2 results of genera with significantly different abundances between Starved and Fed larvae at T24 H and T48 H. A total of 82 genera had significantly (q < 0.05) different abundance between treatments within the T24 H time point, and 64 genera had significant differences in abundances between treatments within the T48 H time point. Highlighted cells indicate the top six genera that were statistically significantly different in abundance across both time points. [file Table_2.DOCX]

| **Supplemental Table 2.** DESeq2 results of genera with significantly different abundances between Starved and Fed larvae at T24 H and T48 H. A total of 82 genera had significantly (*q* < 0.05) different abundance between treatments within the T24 H time point, and 64 genera had significant differences in abundances between treatments within the T48 H time point. Highlighted cells indicate the top six genera that were statistically significantly different in abundance across both time points. | | | | | |
| --- | --- | --- | --- | --- | --- |
| T24 | | | T48 | | |
|  | BaseMean | Adjusted  p- value |  | BaseMean | Adjusted  p- value |
| Act_Actinomyces | 4019.11527 | 0.0000 | Act_Actinomyces | 8984.13074 | 0.0000 |
| Unclassified | 3902.2573 | 0.0000 | Ent_Enterococcus | 1666.102 | 0.0000 |
| Bac_Bacillus | 1904.11902 | 0.0012 | Sph_Sphingobacterium | 1230.24762 | 0.0000 |
| Cam_Campylobacter | 1562.20273 | 0.0006 | Leu_Weissella | 822.12596 | 0.0000 |
| Ent_Enterococcus | 1496.95143 | 0.0084 | Cor_Corynebacterium | 736.580356 | 0.0002 |
| Leu_Weissella | 582.736575 | 0.0000 | Mic_Microbacterium | 631.462712 | 0.0000 |
| Mor_Acinetobacter | 552.593305 | 0.0000 | Cry_Fluviicola | 567.905562 | 0.0000 |
| Sph_Sphingobacterium | 471.710957 | 0.0003 | Mic_Leucobacter | 424.773828 | 0.0000 |
| Mic_Microbacterium | 316.941788 | 0.0005 | Beu_Serinibacter | 387.47177 | 0.0000 |
| Pse_Pseudomonas | 297.182728 | 0.0408 | Lac_Lactobacillus | 308.524365 | 0.0000 |
| Cor_Corynebacterium | 288.901357 | 0.0141 | Bac_Bacteroides | 289.404406 | 0.0000 |
| Lac_Lachnospiraceae_Group | 270.044131 | 0.0000 | Fla_Flavobacterium | 262.354573 | 0.0000 |
| Lac_Lactobacillus | 267.531013 | 0.0024 | Chi_Taibaiella | 224.501664 | 0.0000 |
| The_Pyrobaculum | 255.267629 | 0.0000 | Aer_Facklamia | 223.621129 | 0.0223 |
| Bac_Bacteroides | 231.54125 | 0.0004 | Lac_Lachnospiraceae_Group | 221.018887 | 0.0000 |
| MSP_MSP41_Group | 209.682681 | 0.0000 | Act_Varibaculum | 218.906704 | 0.0005 |
| Cry_Fluviicola | 205.930466 | 0.0008 | Sph_Sphingobacteriaceae_Group | 193.067827 | 0.0000 |
| Beu_Serinibacter | 195.015567 | 0.0000 | Pun_Cerasicoccus | 189.442566 | 0.0000 |
| Lac_Lachnospiraceae | 179.436005 | 0.0000 | Beu_Beutenbergia | 185.005249 | 0.0000 |
| Mic_Leucobacter | 176.426534 | 0.0008 | Rho_Rhodobacter | 174.450488 | 0.0000 |
| Orb_Orbaceae_Group | 159.186686 | 0.0000 | Orb_Orbaceae_Group | 171.140902 | 0.0162 |
| Orb_Gilliamella | 134.098765 | 0.0000 | Hyp_Devosia | 166.994807 | 0.0000 |
| Str_Streptococcus | 133.008656 | 0.0106 | Str_Streptomyces | 166.153317 | 0.0338 |
| Tis_Sedimentibacter | 131.537895 | 0.0000 | Bru_Ochrobactrum | 165.512102 | 0.0001 |
| Act_Varibaculum | 116.023197 | 0.0377 | Lac_Lachnospiraceae | 156.632015 | 0.0000 |
| Cam_Sulfurospirillum | 113.208717 | 0.0001 | Bre_Brevibacterium | 154.247901 | 0.0338 |
| Bac_Peredibacter | 113.041898 | 0.0015 | Rho_Paracoccus | 152.262747 | 0.0000 |
| Str_Streptomyces | 108.137885 | 0.0001 | Str_Streptococcus | 147.128145 | 0.0001 |
| Ent_Vagococcus | 107.882643 | 0.0064 | Act_Arcanobacterium | 124.097116 | 0.0091 |
| Lac_Lachnoclostridium | 105.677373 | 0.0000 | Ent_Vagococcus | 108.731372 | 0.0000 |
| Bru_Ochrobactrum | 104.503907 | 0.0000 | Rho_Rhodobacteraceae_Group | 107.677369 | 0.0000 |
| Lac_Coprococcus | 98.7067539 | 0.0006 | Sph_Parapedobacter | 103.361405 | 0.0000 |
| Beu_Beutenbergia | 96.0953605 | 0.0000 | Act_Trueperella | 100.239526 | 0.0004 |
| Rho_Paracoccus | 70.8641972 | 0.0000 | Sph_Olivibacter | 98.4043155 | 0.0000 |
| Rum_Ruminococcaceae | 70.4770804 | 0.0009 | Der_Dermacoccus | 88.807658 | 0.0164 |
| Cam_Arcobacter | 68.8962768 | 0.0301 | Lac_Lachnoclostridium | 88.7657892 | 0.0000 |
| Orb_Frischella | 67.9379815 | 0.0000 | Ent_Enterobacteriaceae_Group | 88.4213307 | 0.0369 |
| Fer_Fervidicoccaceae_Group | 67.3474249 | 0.0000 | Lac_Coprococcus | 88.3809328 | 0.0051 |
| Rho_Rhodobacter | 67.1780655 | 0.0000 | San_Sanguibacter | 87.4216717 | 0.0021 |
| Str_Lactococcus | 64.9711236 | 0.0242 | Rhi_Agrobacterium | 80.980148 | 0.0015 |
| Beu_Salana | 62.2950651 | 0.0000 | Rum_Ruminococcaceae | 80.2214542 | 0.0369 |
| Lac_Blautia | 57.9704385 | 0.0000 | Str_Lactococcus | 70.2639249 | 0.0000 |
| Car_Granulicatella | 57.0219008 | 0.0027 | Noc_Pimelobacter | 63.3618253 | 0.0003 |
| Hyp_Devosia | 55.9953625 | 0.0000 | Car_Granulicatella | 61.5741684 | 0.0000 |
| Hel_Sulfurovum | 55.8972907 | 0.0000 | Int_Kribbia | 60.8389226 | 0.0164 |
| Lac_Dorea | 54.5161553 | 0.0004 | Lac_Blautia | 60.5422632 | 0.0000 |
| Sph_Sphingobacteriaceae_Group | 54.0384233 | 0.0000 | Alc_Achromobacter | 56.6582189 | 0.0001 |
| Rho_Rhodobacteraceae_Group | 50.2335466 | 0.0000 | Mic_Agromyces | 53.7149583 | 0.0004 |
| Chi_Taibaiella | 48.2424018 | 0.0000 | Cer_Cerasicoccus | 51.8080913 | 0.0000 |
| Lac_Ruminococcus | 43.0473059 | 0.0000 | Gem_Gemella | 48.508043 | 0.0024 |
| Rhi_Agrobacterium | 42.8227247 | 0.0000 | Rum_Ruminococcus | 46.3117186 | 0.0000 |
| Mic_Pseudoclavibacter | 42.4422961 | 0.0011 | Lac_Ruminococcus | 40.8921571 | 0.0000 |
| Cel_Cellulomonas | 42.3035009 | 0.0000 | Lac_Dorea | 40.4114876 | 0.0000 |
| Pun_Cerasicoccus | 41.2294014 | 0.0008 | Xan_Thermomonas | 37.8665214 | 0.0263 |
| Ery_RFN20 | 41.0889966 | 0.0005 | Mic_Herbiconiux | 36.1029724 | 0.0003 |
| Act_Trueperella | 40.419273 | 0.0002 | Phy_Aquamicrobium | 34.5473139 | 0.0003 |
| Rum_Ruminococcus | 40.1883023 | 0.0002 | Pun_Puniceicoccaceae_Group | 32.2582933 | 0.0000 |
| Cau_Brevundimonas | 38.3117997 | 0.0075 | Lac_Roseburia | 31.8495067 | 0.0000 |
| Ent_Citrobacter | 38.1610713 | 0.0171 | Mic_Naasia | 26.635306 | 0.0002 |
| Des_Ignisphaera | 38.0535388 | 0.0000 | Mic_Agrococcus | 26.5866234 | 0.0223 |
| Int_Kribbia | 37.1115272 | 0.0000 | Mic_Lysinimonas | 26.0613448 | 0.0039 |
| San_Sanguibacter | 36.3545619 | 0.0141 | Mic_Chryseoglobus | 22.5298987 | 0.0055 |
| Lac_Roseburia | 32.891122 | 0.0012 | Lac_Anaerostipes | 11.8614886 | 0.0002 |
| Pyr_Pyrodictiaceae_Group | 30.4632109 | 0.0000 |  |  |  |
| Des_Aeropyrum | 29.5313112 | 0.0000 |  |  |  |
| Alc_Achromobacter | 28.4280563 | 0.0222 |  |  |  |
| Fer_Fervidicoccus | 27.7050919 | 0.0010 |  |  |  |
| Mic_Agromyces | 27.6960437 | 0.0010 |  |  |  |
| Noc_Nocardioides | 27.6617582 | 0.0316 |  |  |  |
| Sph_Olivibacter | 27.4937475 | 0.0000 |  |  |  |
| Sph_Parapedobacter | 25.2116239 | 0.0000 |  |  |  |
| Noc_Pimelobacter | 24.2598267 | 0.0084 |  |  |  |
| Der_Dermatophilaceae_Group | 23.9087145 | 0.0006 |  |  |  |
| Phy_Aquamicrobium | 21.5187572 | 0.0000 |  |  |  |
| Lac_Pseudobutyrivibrio | 16.6477944 | 0.0000 |  |  |  |
| Mic_Herbiconiux | 15.28218 | 0.0008 |  |  |  |
| Lac_Anaerostipes | 13.9630627 | 0.0357 |  |  |  |
| Mic_Agrococcus | 13.5452181 | 0.0282 |  |  |  |
| Mic_Lysinimonas | 11.1185765 | 0.0078 |  |  |  |
| Pun_Puniceicoccaceae_Group | 10.5683492 | 0.0156 |  |  |  |
| Mic_Chryseoglobus | 9.01292527 | 0.0255 |  |  |  |
